# School-going transgender youths’ experiences at health care facilities: a systematic scoping review protocol

**DOI:** 10.1186/s13643-020-01347-0

**Published:** 2020-04-24

**Authors:** Delarise M. Mulqueeny, Senzelokuhle M. Nkabini, Tivani P. Mashamba-Thompson

**Affiliations:** 1 Discipline of Public Health Medicine, School of Nursing and Public Health, University of KwaZulu-Natal, Pinetown, South Africa; 2 Department of Social Science, Gender and Education, School of Education, University of KwaZulu-Natal, Room 01-032, 121 Marianhill Rd, Pinetown, 3605 South Africa

**Keywords:** School-going, Transgender, Youth, Experiences, Health care facilities

## Abstract

**Background:**

Globally, miniscule improvements have been implemented regarding equality, inclusion, access, and protection of people with diverse gender identities whilst accessing health care facilities of which transgender youth form part. Literature has highlighted that the care transgender youth receive at health care facilities can result in positive or negative outcomes. School-going transgender youth constitute a unique group whose experiences at health care facilities warrant ongoing research. Hence, the objective of this scoping review is to systematically map evidence of school-going transgender youths’ experiences at health care facilities regarding service delivery, support, and policies and to identify literature gaps that could inform future research.

**Methods:**

We will conduct a scoping review, using peer-reviewed journal articles that present literature on school-going transgender youths’ experiences at health care facilities. Searches for relevant articles will be conducted on the following databases: PubMed, Cochrane Library, Campbell collaboration, Scopus, Embase, and MEDLINE. Additional searches will be conducted on institutional websites or web-based search portals. Two reviewers will independently extract data from all relevant search engines incorporating the study objective, research questions, and eligibility criteria. The inclusion criteria include published full-text qualitative, quantitative, and mixed-method studies that address the topic with no language and publication year limitations to reflect a comprehensive range of literature that includes the implementation of the SDGs. All literature that does not meet the inclusion criteria will be excluded. The quality of included studies will be appraised using the mixed methods appraisal tool (MMAT) – version 2018.

**Discussion:**

We anticipate mapping the experiences of school-going transgender youth at health care facilities. Once summarized, the data could be useful to clinical educators, health workers, policy makers, and guide future research to ensure that the human and patients’ rights of transgender youth, are globally acknowledged, protected, and respected within health care facilities.

## Background

Globally, miniscule improvements have been implemented regarding equality, inclusion, access, and protection of people with diverse gender identities in health care facilities of which school-going transgender youth form part [1–3]. School-going transgender youth refers to individuals attending educational institutions whilst transitioning between childhood and adulthood with their gender identity or expression not matching the sex, they were assigned at birth nor conforming to cisgender norms [4–8]. Many transgender youth, experience non-acceptance and misunderstanding from members of various societies including the medical fraternity while they navigate the complexities and challenges of transitioning between childhood and adulthood [9, 10].

Additionally, members of the transgender population experience and are exposed to a myriad of health challenges and risks such as substance abuse, mental illness, suicide, high mortality, and are at an increased risk of human immunodeficiency virus (HIV) infections [11–13]. They also experience many barriers to patient-centered care when accessing information, treatment, and care at health facilities [14]. These include a lack of gender-affirming policies, the non-execution of gender-affirming practices, health services being conducted according to heteronormative and cis-normative health care frameworks, and the medical curricula excluding the needs of transgender people [15–17]. Additional barriers are few or non-existent gender-affirming medical practitioners and facilities, gender and culturally incompetent health workers having inadequate knowledge to provide gender-affirming service delivery and a lack of or inadequate access to care and medical insurance [18–20]. These barriers can result in transgender individuals having adverse experiences of health care at health facilities [21].

In response to the HIV epidemic, the World Health Organisation (WHO), commissioned a review to identify the most vulnerable populations who are at a high risk of HIV infection [22]. Five (5) key populations (KPs) were identified which include people in prisons, sex workers, people who inject drugs, men who have sex with men, and transgender people [23]. The latter is due to higher-risk behaviors in these populations with 96% of KPs contributing to new HIV infections in Europe, Asia, and Africa [24]. In addition, 90% of new HIV infections in Northern America, Pacific, and Caribbean region (Australia, New Zealand, and surrounding islands) (84%), and Southern America (77%) are attributed to KPs being subordinated or uncomfortable or unable to access health care facilities [24]. Moreover, in response to service delivery, the LGBTQ youth report of 2018 estimated that only 12% of youth who identify as transgender, reported receiving relevant information pertaining to safe sex at health care facilities [3]. Thus, demonstrating a challenge experienced by transgender youth who require relevant information but health care service providers being unable to adequately service their requirements and needs.

The United Nations (UN) general assembly adopted 17 sustainable development goals (SDGs) in 2015 which were initiated in 2016 [25]. These SDGs allow for the inclusion of all members of society through the endorsement of the “leave no one behind” policy which forms part of the 2030 agenda [25]. The SDG 3: Good health and well-being is especially relevant to this review as it addresses universal health coverage for all citizens including people who identify as lesbian, gay, bisexual, transgender, and intersex (LGBTI) [26]. Additionally, this goal addresses the aim to end AIDS and other non and communicable diseases including discrimination and stigma thereby ensuring their health and well-being within countries and health systems [27]. Hence, any inadequate health service delivery or exclusionary practices leveled at transgender individuals deems this goal unaccomplished. Moreover, evidence exists on the global acknowledgement of the SDGs and humanitarian organisations advocacy for the abolishment of violence, transphobia, and other forms of discrimination leveled against transgender people [28, 29].

Our literature searches of the Cochrane database of systematic reviews, Pubmed, and CINHAHL, revealed that several primary studies and systematic and scoping reviews have been conducted on transgender youth in relation to various aspects of health care [30–33]. However, to the best of our knowledge, the last scoping review conducted on transgender youths’ experiences of health and social care was conducted between 2006 and 2016 [34]. This is while many transgender youth still experience extreme human rights violations, gender-based violence and transphobia and are often refused medical care due to their gender identity [35, 36]. Such adverse experiences have social, educational, health, psychological, and societal implications which not only impacts the individual but also their families, societies, and health systems globally [37]. Hence, a scoping review addressing school-going transgender youths’ experiences at health care facilities between January 2015 and February 2020 is relevant as it will include current practice running concurrently with the implementation of the SDGs. It will be beneficial to clinicians, clinical educators, health workers, health departments for service delivery, policies, and practice.

### Objective

To systematically map evidence of school-going transgender youths’ experiences at health care facilities regarding service delivery, support and policies and to identify literature gaps that could inform future research.

## Methods

To effectively map a broad range of information on school-going transgender youths’ experiences at health care facilities, a scoping review was selected. This knowledge synthesis method will employ a phenomenological stance to systematically and rigorously map published literature and identify information gaps on the topic from qualitative, quantitative, and mixed-methods studies [34]. The use of these sources of evidence has previously been used in other scoping reviews relating to “experiences” [34, 38, 39]. The scoping review protocol has not been registered a priori and is guided by Arksey and O’Malley’s five stage scoping review framework, later supplemented by Levac et al. as well as the 2015 Joanna Briggs Institute’s guidelines [40–42]. The five stages highlighted by the framework are (i) identifying the research question, (ii) identifying relevant studies, (iii) selection of eligible studies, (iv) charting the data, and (v) collating, summarizing, and reporting the results. The Preferred Reporting Items for Systematic Reviews and Meta-Analysis: Extension for Scoping Review guidelines (PRISMA-ScR) will provide guidance to ensure this protocol and the review addresses all the steps required [43]. The Preferred Reporting Items for Systematic review and Meta-Analysis Protocols (PRISMA-P) 2015 checklist (Additional file 1) will summarise and present the results [44, 45].

### Identifying the research question

As this review explores school-going transgender youth’s experiences rather than their proxies, caregivers, or health workers only their experiences in their words will be included. Hence, the main research question is as follows:

What is known about school-going transgender youths’ experiences at health care facilities?

The secondary research questions are as follows:

- What is known about the factors (service delivery, support, and policies) that contribute to school-going transgender youths’ experiences at health care facilities?
- What is known about the consequences of school-going transgender youths’ experiences at health care facilities?

#### Eligibility of the research question

A PCC (**p**opulation, **c**ontext, **c**oncept) framework (Table 1) will adequately address the eligibility of the research question [42].

**Table 1:** PCC (**p**opulation, **c**ontext, **c**oncept) framework

Criteria	Determinants
**P**opulation	**School-going transgender youth** **School-going** refers to individuals that are children, adolescents, and youth that attend an institution for basic education [4]. **Transgender** refers to individuals whose gender identity or expression does not match the sex they were assigned at birth [5]. **Youth** refers to the phase between childhood and adulthood [6].
**C**oncept	**Experiences** in this review, utilizes The Beryl Institute’s definition of “patient experiences” and includes “the sum of all interactions, shaped by an organization’s culture, that influence patients’ perceptions, across the continuum of care” [7].
**C**ontext	**Health care facilities** refer to institutions that provide care or improvement of health through prevention, diagnosis and treatment of disease, illness, injury, and other physical and mental impairments affecting people [8].
**Publication Year Range:** None	**Language:** All

### Identifying relevant studies

Searches will be conducted for evidence published in peer-reviewed journals from the following databases and libraries: PubMed, Cochrane Library, Campbell collaboration, Scopus, Embase, and MEDLINE to attain a wide range of relevant articles and literature. Additional searches will be conducted in the following databases: EBSCOhost, Google Scholar, WHO, UNFPA, UNICEF, UNAIDS, UNESCO, UNDP, UNODC, USAID, PEPFAR, and IRGT. The search strategy will be informed by key terms including: “school-going”, “transgender youth”, “experiences”, and “health care facilities”. The search strategy will be guided by a public health librarian. Studies written in all languages with no publication date restriction will be selected to reflect older and current practices. This is to attain a comprehensive literature range that also includes the impact of the SDGs on school-going transgender youths’ experiences at health care facilities. A hand search of included articles reference lists will be conducted by a research assistant to obtain additional literature. Corresponding authors will be contacted by the research assistant to attain inaccessible articles. To test the selected keywords and databases and reduce and eliminate any bias, two pilot searches were conducted by two (DMM, SMN) screeners. The results thereof are presented in Table 2.

**Table 2:** Pilot database search results

Keywords	Date of search	Name of search engine	Number of publications
((“transgender persons”[MeSH Terms] OR (“transgender”[All Fields] AND “persons”[All Fields]) OR “transgender persons”[All Fields] OR “transgender”[All Fields]) AND Experiences[All Fields]) AND (“health facilities”[MeSH Terms] OR (“health”[All Fields] AND “facilities”[All Fields]) OR “health facilities”[All Fields] OR (“health”[All Fields] AND “care”[All Fields] AND “facilities”[All Fields]) OR “health care facilities”[All Fields])	22/02/2020	PubMed	26
“School-going”	22/02/2020	Cochran Library	126
“School and transgender persons”			16
“Transgender”			246
“Transgender Youth”			20
“Transgender experiences”			18
“Transgender health-care facilities”			2
			Total: 428

### Study selection

The selection of eligible studies will be based on the title, abstract, content relating to school-going transgender youths’ experiences at health care facilities, the research question, and inclusion and exclusion criterion. An Endnote X9.2 library will be exclusively utilized to upload relevant literature for this scoping review. All duplicate studies will be deleted prior to the screening process commencing. Comprehensive title and abstract screening will be independently conducted in parallel by the primary author (DMM) and co-screener (SMN). All disagreements at this stage will be resolved through discussion and a third screener (TMT). Thereafter, full article screening will also be independently conducted by two screeners (DMM, TMT) with a third reviewer (SMN) resolving discrepancies during this stage. Excluded sources of evidence, due to them not meeting the inclusion criteria, will be documented in an additional file with the rationale for their exclusion. This is to assist with reproducing this study.

#### Inclusion and exclusion criteria

**Fig. 1:**
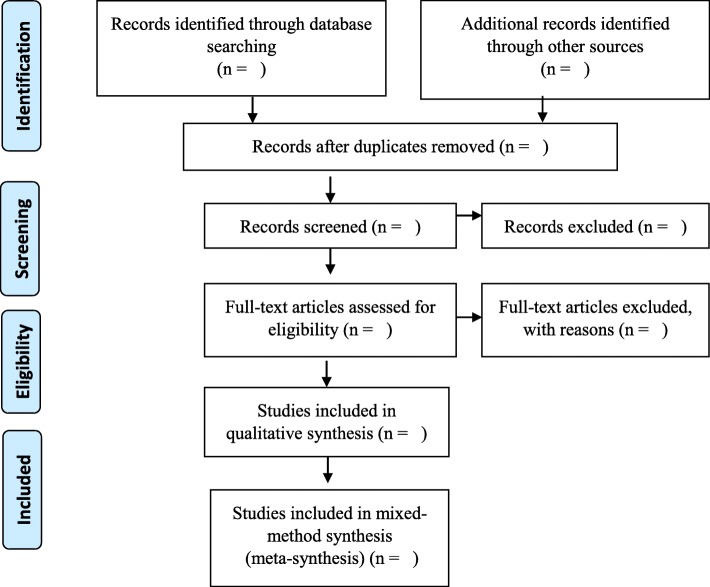
PRISMA ScR flow chart which demonstrates literature searches and sources of evidence selections for school-going transgender youth experiences of health care facilities [43]

Included studies will meet the following criteria: (i) focus on school-going transgender youths’ experiences at health care facilities, (ii) be full-text qualitative, quantitative, and mixed-method studies (iii) have no language or publication year range restrictions. Studies that do not focus on school-going transgender youths’ experiences at health care facilities and are not available in full-text will be excluded. Commentaries, observational studies, white papers, editorials, opinion pieces, and unpublished thesis that do not contain quotes from school-going transgender youth will also be excluded. The PRISMA ScR flow chart captures and presents our planned screening and selection process (Fig. 1).

### Charting the data

Two screeners (DMM, SMN) developed a data extraction form (Table 3) in a Google form which will be piloted by (DMM, TMT). For the actual review, extracted data from included sources of evidence will be independently charted and transferred to the form by two screeners (DMM, SMN) while another screener (TMT) will independently confirm appropriateness for their inclusion. As this is an iterative process, all the screeners will meet after charting 10 articles to review the process and ensure that all the variables meet the requirements of the review. The PRISMA ScR will constantly be updated by the research assistant.

**Table 3:** Data extraction form

1. **Authors names and date**	.
2. **Study title**	
3. **Study aim**	
4. **Study design**	
5. **Study setting**	
6. **Study population** - **Age** - **Grade**	
7. **Population quotations**	
8. **Health conditions**	
9. **Type of encounter (chronic or acute)**	
10. **Significant findings**	
11. **Conclusions**	
12. **Notes**	

### Collating, summarizing, and reporting the results

As this scoping review includes various study designs, this process comprises of three stages (a) descriptive, thematic, and quantitative analysis (b) reporting the results, and (c) identifying literature gaps for future research. The qualitative analysis will contain quotations and be conducted by all three screeners incorporating Braun and Clarke’s thematic framework and the use of the NVivo data analysis software [46]. The quantitative analysis and graphical illustrations will be independently conducted by two screeners (DMM, SMN) utilizing the Microsoft Excel program. The themes from the extracted data will be examined in relation to, the objective of the study which is to map evidence of school-going transgender youths’ experiences at health care facilities, the validity of the research question and the suggestions from the findings for future research. Daily reflexive meetings and consensus discussions will eliminate any bias, and discrepancies will be resolved by consensus between the primary author and other screeners throughout the process [47].

#### Quality assessment

The mixed methods appraisal tool (MMAT) – version 2018 will be used to assess the quality of included evidence to ensure that the study objective is rigorously presented and to eliminate any risk of bias [48]. Quality and elimination of partiality will be achieved by examining author names and date, title, aim of study, study design, study setting, study population, population age and grade, concept, significant findings, key conclusions and notes. The MMAT guidelines (100% high average, 75% above average, 50% average, 25% low quality) will be used to rate included articles. The quality of all included articles will be presented in a table, so readers know how to approach the various articles.

## Discussion

The proposed scoping review will generate findings that identify and describe the experiences of school-going transgender youth while accessing services at health care facilities and identify knowledge gaps. As a scoping review methodology with a phenomenological slant is embraced, cognizance must be taken of the limitations, and strengths of adopting an interpretive epistemological stand [34, 49]. The strengths of the review are its unique synthesis of school-going youths’ experiences from direct quotations and that the results can be transferable in a broad context as eligible studies are not limited by geographical location nor health encounter. Additionally, the iterative and explorative nature of the review strengthens its rigor [50]. The experiences of the population under review are relevant as the United Nations 2019 estimates revealed that youth account for approximately 16% of the global community [51]. The review will present balanced data as it will contain literature relating to their successful and challenging experiences which have frequently been silenced when assessing health care outcomes. Moreover, it facilitates the importance of patient-centered care through the acknowledgement of patients’ experiences which could highlight strategies to improve health systems, facilities, education, and service delivery to this key population [52]. The findings can also make a valuable contribution to ensuring that the human and patients’ rights of transgender youth are acknowledged, respected, and protected within health care facilities. Additionally, this review focusses on an area that is relevant and beneficial to transgender patients, policy makers, clinicians, health educators, and health care workers. Humanitarian organizations as well as funders could find the results helpful to fund evidence-based interventions aimed at promoting patient-centered care and eliminating transphobia within health care settings.

The outcomes of this scoping review will be published in peer-reviewed journals and presented at international conferences, seminars, and symposiums. The presentation of the findings will assist in addressing the role of governments, community-based organizations, health education, policies and awareness campaigns pertaining to gender identity, transphobia, patient-centered care, and discrimination on a global scale.

## Limitations

A scoping review with no language or time limitations could present financial and time challenges that require stringent timelines and monitoring.

## Supplementary information

**Additional file 1.** PRISMA-P 2015 Checklist.

## Data Availability

All data generated or analyzed during this study will be included in the published scoping review article and will be available upon request.

## Abbreviations

HIV: Human immuno-deficiency virus
IRGT: A global network of trans women and HIV
KP: Key populations
LGBTI: Lesbian, gay, bisexual, transgender, intersex
LGBTQ: Lesbian, gay, bisexual, transgender, queer
PEPFAR: The United States Presidents Emergency Plan for Aids Relief
PRISMA: Preferred Reporting Items for Systematic and Meta Analyses
PRISMA-P: Preferred Reporting Items for Systematic review and Meta Analyses Protocols
PRISMA-ScR: Preferred Reporting Items for Systematic Reviews and Meta-Analyses Extension for Scoping Reviews
SDG: Sustainable development goals
UNAIDS: The Joint United Nations Programme on HIV/AIDS
UNESCO: United Nations Educational Scientific Cultural Organization
UNDP: United Nations Development Plan
UNODC: United Nations Office on Drugs and Crime
USAID: United States Agency for International Development
WHO: World Health Organisation
